# Supplementary material for: Evidence-based policy-making in sports funding using a data-driven optimization approach
Source: PLoS One. 2024 Dec 31;19(12):e0312179. doi: 10.1371/journal.pone.0312179 (PMC11687695; doi:10.1371/journal.pone.0312179)
Supplement: Attachment — Submitted filename: Cover_Letter_sport_abm_revision.pdf [file pone.0312179.s001.pdf]

Dear Editor,

We sincerely appreciate the time and effort you and the reviewers invested in evaluating our manuscript. Their insightful feedback has been invaluable in refining our work. In response to the reviewers' comments, we have made substantial revisions to enhance the clarity and robustness of our manuscript. The key improvements include:

1.PLOS ONE Template and Style Requirements:

We have formatted the manuscript according to the PLOS ONE LaTeX template and ensured that all elements follow the style guide provided in the links. This includes adherence to file naming conventions and other formatting standards.

2.Figures and Copyright

The maps in Figures 1 through 5 were generated using our own scripts and datasets. As such, these maps are under our own copyright, and no third-party data or copyrighted content has been used. Therefore, there are no copyright issues related to these figures, and they are fully compatible with the CC BY 4.0 license.

3.Funding Information

We have updated the financial disclosure statement to comply with the PLOS ONE guidelines. The updated statement includes the specific grant numbers, initials of the author who received each award, full names of the funding agencies, URLs to the sponsors' websites, and a statement about the funders' role in the research. The revised financial disclosure statement is as follows:

> **Financial Disclosure Statement**
> The project was funded by the European Union's Structural Reform Support Programme (SRSP) under Grant Agreement No. GA2020/023 (received by S.T.; `https://commission.europa.eu/about-european-commission/departments-and-executive-agencies/structural-reform-support/structural-reform-support-programme-srsp_en`), and the Austrian Research Promotion Agency (FFG) under project number P 882184 (received by S.T.; `https://www.ffg.at/en`). The funders had no role in study design, data collection and analysis, decision to publish, or preparation of the manuscript.

Thank you for considering our revised manuscript.

Best regards,

Peter Klimek, PhD

Assoc. Prof. Medical University of Vienna
Complexity Science Hub Vienna

Response to reviewer #1

1. The topic is contextualized in the Introduction section to physical inactivity and obesity. The covid-19 pandemic is also mentioned. This frame is not discussed much in Discussion or elsewhere in the manuscript after the Introduction (or Abstract). At the very beginning, a hypothesis is even being developed between the pandemic, obesity and low physical activity. I would suggest that this framework will be removed and research should focused on how public sports funding should be targeted as a sport policy activity. The factors for that are the size of the clubs, funding, socio-economic status, etc. This is also related to the very important issue brought up in Discussion/Conclusion, i.e. knowledge based management and information/data as tool in political decision-making.

We thank the reviewer for this valuable comment. We have carefully considered the suggestion and agree that the focus of the manuscript should be more clearly aligned with the targeting of sports funding as a policy activity. As such, we have revised the Introduction section to remove the references to COVID-19 and obesity. The updated Introduction now focuses on the key factors relevant to sports funding policy, such as the size of sports clubs, funding allocations, and socio-economic status.

2. In the Conclusions/Discussion section, observations related to the material are highlighted. They are relevant. I suggest that, in the same context, authors also consider the possibilities of scaling this research to sports other than football, or possibly to the funding of non-organized sports that try to promote the movement of the population.

We thank the reviewer for this suggestion. We considered expanding the research to include other sports; however, due to the unavailability of data on the number of children participating in sports clubs besides football, this was not feasible at this time. Our current dataset includes detailed information only for football clubs. Without similar data for other sports, it would be difficult to apply the same methodological framework. Nevertheless, we acknowledge that scaling the research to other sports or even non-organized sports activities would be a valuable future direction.

3. I also suggest that the grammar of the manuscript will be checked. The text is mostly at a good level, but sentence structures and general fluency can be improved.

We thank the reviewer for this suggestion. We have carefully proofread the manuscript and made several revisions to improve fluency and readability.
